# Synovium-Derived Mesenchymal Stem Cell Transplantation in Cartilage Regeneration: A PRISMA Review of *in vivo* Studies

**DOI:** 10.3389/fbioe.2019.00314

**Published:** 2019-11-15

**Authors:** Kendrick To, Bridget Zhang, Karl Romain, Christopher Mak, Wasim Khan

**Affiliations:** ^1^Division of Trauma and Orthopaedics, Department of Surgery, Addenbrooke's Hospital, University of Cambridge, Cambridge, United Kingdom; ^2^School of Clinical Medicine, University of Cambridge, Cambridge, United Kingdom

**Keywords:** mesenchymal stem cells, synovium, transplantation, cartilage repair, osteoarthritis

## Abstract

Articular cartilage damaged through trauma or disease has a limited ability to repair. Untreated, focal lesions progress to generalized changes including osteoarthritis. Musculoskeletal disorders including osteoarthritis are the most significant contributor to disability globally. There is increasing interest in the use of mesenchymal stem cells (MSCs) for the treatment of focal chondral lesions. There is some evidence to suggest that the tissue type from which MSCs are harvested play a role in determining their ability to regenerate cartilage *in vitro* and *in vivo*. In humans, MSCs derived from synovial tissue may have superior chondrogenic potential. We carried out a systematic literature review on the effectiveness of synovium-derived MSCs (sMSCs) in cartilage regeneration in *in vivo* studies in accordance with Preferred Reporting Items for Systematic Reviews and Meta-Analyses (PRISMA) protocol. Twenty studies were included in our review; four examined the use of human sMSCs and 16 were conducted using sMSCs harvested from animals. Most studies reported successful cartilage repair with sMSC transplantation despite the variability of animals, cell harvesting techniques, methods of delivery, and outcome measures. We conclude that sMSC transplantation holds promise as a treatment option for focal cartilage defects. We believe that defining the cell population being used, establishing standardized methods for MSC delivery, and the use of objective outcome measures should enable future high quality studies such as randomized controlled clinical trials to provide the evidence needed to manage chondral lesions optimally.

## Introduction

Damage to articular cartilage can occur as a consequence of trauma or disease (Thomas et al., 2017). Cartilage is a relatively avascular structure, and has a limited ability to repair (Convery et al., 1972). In an attempt to do so, inflammation ensues within the joint with long term sequelae including osteoarthritis (Soren et al., 1976; Lohmander and Roos, 2007). Musculoskeletal disorders including osteoarthritis are the most significant contributor to disability globally (GBD 2015 Disease and Injury Incidence and Prevalence Collaborators, 2016), and arthritis alone affects over 8 million people in the UK (National Collaborating Centre for Chronic Conditions (UK), 2008). Osteoarthritis adversely affects joint function and causes long term pain (Jordan et al., 2009). Management options for focal chondral lesions and its associated consequence such as osteoarthritis are focused on symptom control or establishment of a non-progressive state (Felson et al., 1995). Options such as autologous chondrocyte implantation (ACI) have been explored for focal chondral lesions, but they come with a variable success rate and are associated with complications such as donor-site morbidity (McCarthy et al., 2016). Other treatment options such as acellular biomaterial implantation are resource intensive and are associated with a high failure rate (Buma et al., 2003). The end-stage treatment of osteoarthritis is a joint replacement but this is costly and can have a poor outcome (Lenza et al., 2013). This incurs a significant financial burden that is growing due to an aging population and greater patient expectations.

One way to address the challenge of managing chondral lesions may be through cell-based regenerative therapies. Mesenchymal stem cell (MSC) transplantation is gaining attention as a potential treatment option that reverses chondral lesions (Trounson and McDonald, 2015). MSCs are multipotent stem cells present in various sites of the body including the bone marrow, dental pulp, adipose tissue, synovium, and umbilical cord (Wexler et al., 2003; Lee et al., 2004; Ghorbani et al., 2014; Hatakeyama et al., 2017). As MSCs can be harvested from various tissues, many studies are now focused on ascertaining the optimal cell source that provides the greatest number of cells with the greatest chondrogenic potential (Ronzière et al., 2010; Davies et al., 2017). There is some evidence to suggest that the tissue type and anatomical site from which MSCs are harvested play a role in determining their ability to regenerate cartilage (Pizzute et al., 2015; Hatakeyama et al., 2017). There is evidence to suggest that synovium-derived MSCs (sMSCs) may have superior chondrogenicity in humans (Ogata et al., 2015), and they may prove to be the optimal source cell as they are native to the joints they are targeting. There are no cell surface markers unique to sMSCs, and characterization is based on generic epitopes of MSCs such as CD34, CD35, CD73, CD90, and CD105 (Hermida-Gómez et al., 2011). The *in vitro* chondrogenic differentiation of MSCs depends on exposure to appropriate culture medium including TGF-β3 and glucocorticoids (Derfoul et al., 2006; Bian et al., 2011). Type II collagen (COLII), aggrecan (ACAN), and Sox9 gene expression allows for quantification of chondrogenicity in MSCs *in vitro* (Akiyama et al., 2002; Mwale et al., 2006; Tiruvannamalai Annamalai et al., 2016). Recent studies have demonstrated that sMSCs exhibit greater expression of some of these markers when compared to other MSCs e.g., bone marrow-derived MSCs (Ogata et al., 2015). MSCs may be transplanted autologously, allogenically, or xenogenically.

It is suggested that MSCs mediate their chondrogenic effects through direct or indirect mechanisms. Articular MSCs within physiological joints demonstrate some ability to exert an endogenous response to cartilage injury (Baboolal et al., 2016). It has been shown through imaging studies that transplanted MSCs are able to migrate to injured joints (Wood et al., 2012; Maerz et al., 2017). However, resident cells are low in numbers and typically go on to exhibit exhaustion followed by senescence when full-thickness chondral lesions are incompletely repaired (Fellows et al., 2017). Furthermore, MSC populations can diminish as a function of age and disease (Asumda and Chase, 2011; Alt et al., 2012). Therefore, it may be beneficial to introduce exogenous MSCs that can directly repair cartilage by producing hyaline cartilage (Zhang et al., 2017) or by acting as a stimulus for chondrogenic cells in addition to native MSCs. MSCs are able to induce differentiation of chondroprogenitors to chondrocytes through secretion of growth factors such as Transforming Growth Factor β (TGF-β) and Fibroblast Growth Factor (FGF) (Ng et al., 2008; Schinköthe et al., 2008) MSCs also secrete prostaglandins (PGE) which subsequently increase Interleukin-10 (IL-10) and decrease IL-12 secretion by dendritic cells (Beyth et al., 2005; Saldaña et al., 2019). This has been shown to promote a T-cell class switch from a pro-inflammatory Th1 to an anti-inflammatory Th2 subtype (Beyth et al., 2005). This could be a mechanism through which MSCs prevent inflammatory joint disease progression. MSCs have also demonstrated the ability to transduce signals via extracellular vesicles (EV) (Baglio et al., 2015). EV released by MSCs have been shown to promote type II collagen deposition in chondral lesions (Wang et al., 2017). EVs that contain miR-140-5p are able to stimulate chondrocyte proliferation and migration to sites of chondral lesion (Tao et al., 2017). This trophic effect may be a mechanism through which MSCs are translocated to sites of injury. Furthermore, it has been shown that bone marrow-derived human MSCs secrete hyaluronan-coated EVs that contain mRNA for CD44 (Arasu et al., 2017).

The transplantation of MSCs is now being tested in over 300 registered clinical trials (Trounson and McDonald, 2015). In this PRISMA systematic review, we examine the potential for sMSCs to regenerate cartilage by analyzing *in vivo* studies in the literature.

## Materials and Methods

A systematic review of the literature was performed in accordance with Preferred Reporting Items for Systematic Reviews and Meta-Analyses (PRISMA) guidelines (Moher et al., 2015). A literature search from conception to January 2019 was performed using PubMed, EmBase, Scopus, and Medline. The following search terms were used: (((((synovial) OR synovium)) AND (((mesenchymal stem cell) OR MSC) OR stem cell)) AND (((((cartilage) OR tendon) OR chondral) AN D (((repair) OR regenerate) OR regeneration). Inclusion and exclusion criteria were applied to the results of the search as follows:

Inclusion Criteria:

1. All articles in the English language with full text available.
2. Articles examining sMSCs using *in vivo* experiments.
3. Articles examining animal and human subjects.
4. Articles with subjects regardless of age, gender, race and pre-treatment health.
5. Articles which examined autologous, allogenic and xenogenic transplantation methods.

Exclusion Criteria:

1. Articles which were not translated into English language and did not have full-text available.
2. Articles that conducted *in vitro* experiments exclusively.
3. Articles that investigated the repair of meniscal lesions were excluded.

KT, BZ, and KR applied the search with above criteria independently. A risk of bias analysis was carried out using Cochrane's tool; Risk of Bias 2.0 (RoB 2). This was performed by BZ and KR independently. Each study was allocated a low, intermediate or high risk of bias in accordance with the RoB 2.0 guidance in the five categories as shown in Figure 3. Various signaling questions (Sterne et al., 2019) (Supplementary Table 1) were answered in each category, the outcomes of the signaling questions were averaged to produce an overall risk in each of the five categories. An overall risk of bias was then determined for each study by the cumulative result of these five categories. A study was judged to be at high risk of bias if it was at high risk for at least one category, or had some concerns for multiple categories. A study was judged to be of some concern if there were some concerns in at least one category, but not to be at high risk in any category. A study was judged to be at low risk of bias if at low risk of bias for all five categories. The summary of the results as a percentage of all the studies is represented in Figure 2. The final articles were reviewed in full text for qualitative synthesis by KT.

## Results

Three-hundred and fifty-four articles were found from PubMed. An additional 35 articles were retrieved from three other sources. No duplicates were identified. A total of 389 articles were retrieved, the title and abstract of each article was screened for appropriateness. Three-hundred and twenty six articles were removed by application of the exclusion criteria.

**Figure 1:**
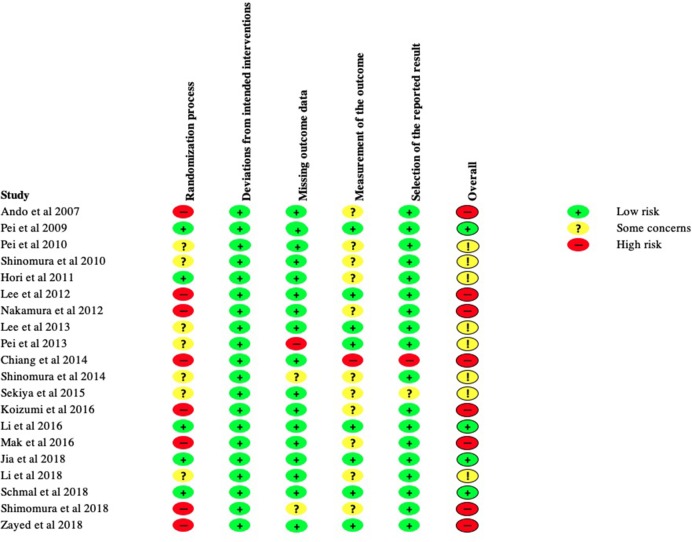
Flow diagram illustrating search process.

Sixty-three full-text articles were reviewed. A total of 20 studies were obtained as shown in Figure 1. A total of 20 studies were included in qualitative synthesis in this. Four out of 20 studies investigated human sMSCs and the remaining studies assessed animal sMSCs.

**Figure 2:**
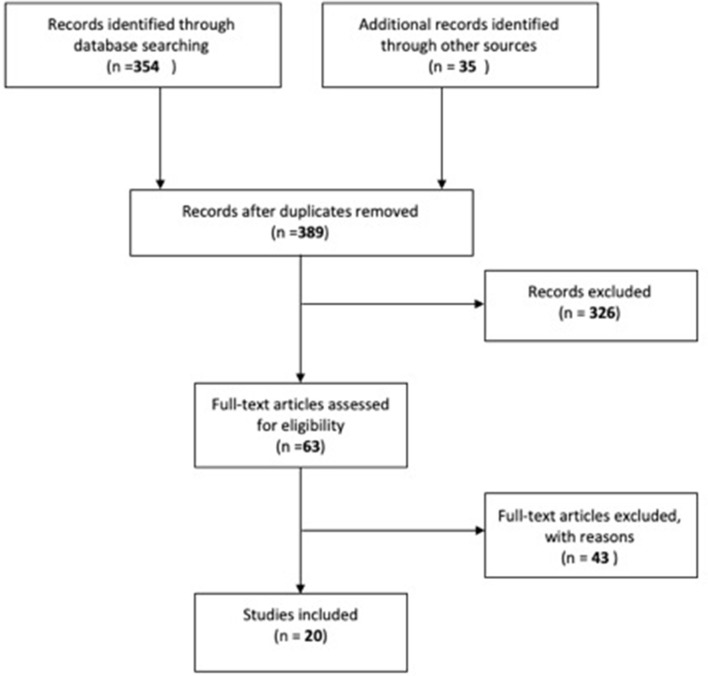
Summary of overall bias.

The outcome of risk of bias analysis is summarized in Figure 2. Twenty percentage of the studies in our review were deemed to have an overall low risk of bias. Forty percentage of the studies were thought to demonstrate high risk of bias and the remainder were thought to demonstrate moderate risk of bias.

**Figure 3:**
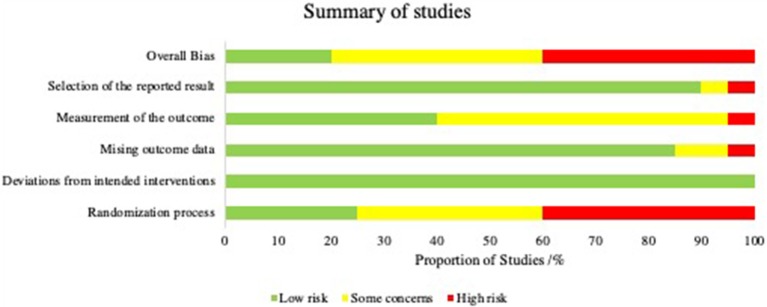
Risk of bias in individual studies.

### Studies of Human sMSCs

Table 1 includes details of the four *in vivo* studies that utilized human sMSCs; two were case-control studies and two were case series.

**Table 1:** Studies of human synovium derived MSC in the repair of cartilage *in vivo*.

**References**	**Study design**	**Cell source**	**Subject**	**Number of subjects**	**Number of controls**	**Method of extraction**	**Cell treatment**	**Method of delivery**	**Outcome**
Li et al. (2018)	Case control	Human	Murine	10	10	Arthroscopic flushing fluid from knee joint	Flushing fluid cells were plated on culture medium. Colony forming assays, flow cytometry, and proliferation assays were used to validate MSCs. MSCs were induced in chondrogenic medium and subsequently fixed and stained with Safranin O	Xenogenic injection of MSC encapsulated in polyPEGDA/HA hydrogel into full thickness cartilage defects in trochlear groove	Reduction in defect area compared to control at 4 and 8 weeks
Shimomura et al. (2018)	Case series	Human	Human with knee OA	5	N/A	Arthroscopic biopsy	Cultured cells were characterized by flow cytometry for MSC markers. They were not induced into chondrogenic differentiation	Autologous implantation of cultured tissue-engineered construct (TEC) into chondral defects without fixation	Improvement in PROMs for pain, ADL, QoL, at 48 months. Secure defect filling confirmed by second look arthroscopy, improved MRI score
Sekiya et al. (2015)	Case series	Human	Human	10	N/A	Arthroscopic biopsy of subsynovial tissue on the femur at the suprapatellar pouch	Synovial MSCs were cultured with Invitrogen containing antibiotics. No *in vitro* assays were carried out to assess chondrogenic differentiation	Autologous injection of cultured cells into femoral condyle defect	Improved qualitative appearance in cartilage defect filling in four out of 10 patients, improved MRI score and increased Lysholm score at an average 52 month follow up
Koizumi et al. (2016)	Case control	Human	Murine	36 knees in 18 rats	4 knees in 2 rats	Arthroscopic biopsy of synovium from knee joint of patients with rheumatoid arthritis or with osteoarthritis or without either	Synovial MSCs were cultured in chondrogenic medium and evaluated. Total RNA from synovial MSC pellets were analyzed using Real Time PCR. qRT-PCR was also used to assess gene expression. GAG expression was quantified using a protein assay.	Xenogenic transplantation of MSC on a tissue engineered scaffold	Osteochondral repair using MSC derived from all patients were superior to control. There was no difference between cells from RA, OA, or normal patients

All human studies used an arthroscopic approach to the knee in order to harvest sMSCs. Li et al. (2018) isolated sMSCs from arthroscopic flushing fluid. The three other studies utilized samples of synovial tissue from arthroscopic biopsies of the knee joint. Two studies examined the effects of autologous sMSC implantation following *ex vivo* expansion. The other two studies carried out xenogenic transplantation and examined the effect of human sMSC on murine models. The method of sMSC delivery varied between the studies. All studies found that sMSCs, when implanted into a chondral lesion, was superior in repairing the lesion as compared with the various different controls or untreated groups.

In the study by Li et al. (2018), human sMSCs from arthroscopic flushing fluid were expanded *ex vivo* before undergoing flow cytometry to analyze cell surface marker expression (Li et al., 2018). Subsequently, one third of the cells were subject to chondrogenic differentiation. Strong expression of proteoglycans along with positive Safranin O immunostaining was observed at 2 weeks following induction. The cells were then encapsulated in hydrogel to form a composite method of delivering sMSCs. The composite was subsequently injected into full thickness chondral lesions in the trochlear groove of the rat femur in 10 mice. Ten other mice were treated with hydrogel alone without the sMSCs and another 10 served as a control. The lesions were reassessed at 4 and 8 weeks following treatment. Macroscopically, the group treated with hydrogel and sMSC demonstrated a greater degree of defect filling. An objective quantification of the macroscopic appearance as per the International Cartilage Repair Society (ICRS) criteria confirmed this. On histological analysis, the sMSC-treated group filled the lesion with tissue that positively stained for Safranin O. Notably, the lesions were partly filled with hydrogel treatment alone, albeit this was reveal to be predominantly due to fibrous tissue forming in the absence of cartilage. No overt complications were observed in the murine subjects.

Koizumi et al. (2016) sought to assess whether source cells from patients with rheumatoid arthritis (RA) or osteoarthritis (OA) would differ in therapeutic potential. The disease cohort comprised of patients who had undergone total knee arthroplasty, arthroplasty of the forefoot and synovectomy of the hand as a result of RA or OA. The sMSCs were xenogenically implanted on a tissue engineered scaffold into rat femoral trochlear groove osteochondral lesions. The *in vitro* experiments showed no significant difference between the groups in chondrogenic potential and cytokine expression. Treated lesions showed higher histological scores compared with untreated groups and there were no differences between the RA and OA groups.

Shimomura et al. (2018) performed human autologous sMSC transplantation in five patients with 1.5–3.0 cm^2^ symptomatic chondral knee lesions. Synovial tissue was obtained through biopsy using an arthroscopic approach and sMSCs were subsequently cultured and characterized *in vitro* prior to transplantation. Immunohistology analysis was carried out and the cells were subsequently transferred onto a tissue engineered construct before implantation. The constructs were implanted into the defect site without fixation. The patients were follow-up at intervals up to 48 weeks post-operatively. Magnetic resonance imaging (MRI) was utilized to assess the chondral lesions. All patients achieved full defect filling at 48 weeks post-intervention as assessed by MRI. While no adverse effects were recorded in the long term, all subjects had mild joint symptoms of pain that resolved within 4 weeks. Some tissue edema was visualized on MRI around the tissue construct at 6 weeks and at 24 weeks. This was found to have resolved by 48 weeks for all cases. Tissue integration and chondrogenesis were assessed histologically. The regenerated tissue stained strongly for Safranin O. No adverse clinical events were reported. The patients reported significant clinical improvement at final 24 month follow-up.

Sekiya et al. (2015) explored the use of human sMSC implantation in a symptomatic single femoral condyle chondral lesion in 10 patients. Five patients underwent concomitant procedures including ACL repair. Following isolation and expansion of sMSCs in 10% autologous human serum for 14 days, a volume of 0.5 mL was implanted into the chondral lesion using a needle connected to a 10 mL syringe. The qualitative appearance was improved on second-look arthroscopy. There was also an improvement in MRI score from 1.0 pre-intervention to 5.0 post-intervention (*p* = 0.005). The histological appearance of the lesions demonstrated the presence of hyaline cartilage and fibrous cartilage. The patients were also assessed clinically and the Lysholm score increased after treatment whereas the Tegner Activity Level Scale did not decrease.

### Studies of Animal sMSCs

Sixteen studies assessed the use of animal derived sMSCs, and were conducted between 2007 and 2018 (Table 2).

**Table 2:** Studies of MSCs derived from animal synovium in the repair of cartilage *in vivo*.

**References**	**Cell source**	**Subject**	**Number of subjects**	**Number of controls**	**Method of extraction**	**Cell expansion**	**Method of delivery**	**Outcome**
Schmal et al. (2018)	Rabbit	Rabbit	6	6	Excisional biopsy of synovium from knee of rabbit	MSCs were cultured in chondrogenic media for 21 days. Chondrogenic differentiation was evaluated through RNA analysis using qPCR	Allogenic transplantation of sMSC into full-thickness cartilage lesions in central medial femoral condyle	Improved ICRS in sMSC group at up to 24 weeks, improved macroscopic appearance
Pei et al. (2009)	Rabbit	Rabbit	12	12	Synovial tissue from knee joint O	Passage 3 synovial MSCs were attached onto PGA mesh and incubated in a bioreactor containing growth factors for 4 weeks. The samples were analyzed with immunohistology and western blot to confirm chondrogenic differentiation	Allogenic implantation of sMSCs on cell-engineered tissue construct into full thickness femoral condyle cartilage defect	Improved qualitative appearance of cartilage defect at 6 months
Lee et al. (2013)	Rabbit	Rabbit	27	27	Excisional biopsy of infrapatellar fat pad	Synovial MSCs were suspended in chondrogenic culture medium, histological analysis was utilized to determine GAG expression and immunohistology was used to determine Collagen II expression at 4 weeks after cultivation	Allogenic transplantation of synovial membrane derived MSCs supported by platelet rich plasma (PRP) into osteochondral defect in trochlear groove of femur	Improved qualitative macroscopic appearance and histological findings
Shimomura et al. (2014)	Rabbit	Rabbit	23	18	Excision of synovial membrane from knee joints of rabbits	Synovial MSCs were suspended in growth medium containing DMEM and FBS. Cells at passage 3–7 were utilized	Allogenic implantation of combined implant made of scaffold-free tissue engineered construct from MSCs and Hydroxyapatite artificial bone into a osteochondral defect of femoral groove	Improved histological scores and improved macroscopic appearance at 1, 2, and 6 months
Li et al. (2016)	Rabbit	Rabbit	5	5	Excision of synovial tissue from knee joint	Synovial MSCs were isolated and incubated in culture flasks containing chondrogenic medium. The cells were not assessed for chondrogenic differentiation	Allogenic injection of MSC into full-thickness cartilage defect in central portion of femoral trochlea groove	No significant difference in MSC vs. control group in macroscopic and MRI scores. Improvement in tissue quality was observed by MRI
Lee et al. (2012)	Rabbit	Rabbit	20	20	Synovial tissue from knee joint	Second passage synovial MSCs were trypsinized and suspended in a composite gel containing collagen, hyaluronic acid, and fibrinogen. The cells were then cultured on a plate with chondrogenic medium. Proliferation assay, RT-PCR, Real Time PCR, and GAG staining were used to assess chondrogenic differentiation	Allogenic injection of SDCS in a composite gel into full thickness defect in the patellar groove of the distal femur	Improved macroscopic scores for Gel SDSC group vs. control, increased immunostaining
Jia et al. (2018)	Rabbit	Rabbit	6	6	Synovial fluid from knee joint obtained through arthrocentesis	Cells were isolated from pellets which were formed by centrifugation of synovial fluid. The cells were culture expanded and induced by chondrogenic differentiation medium. Histological staining and qRT-PCR were used to quantify chondrogenic marker expression e.g., Col2A1, Sox9, and LPL	Allogenic intra-articular injection into cartilage defects of the patellar groove of femur weekly for 4 weeks	Improved ICRS scores in predifferentiated chondrogenic MSCs treated compared to control groups, a third undifferentiated MSC group was also used and was found to be superior to predifferentiated group in ICRS macroscopic score at 12 weeks
Hori et al. (2011)	Murine	Murine	24	24	Excision of synovial membrane at medial femoral condyle	Harvested synovium MSCs were expanded on plates with culture medium. Passage 3 cells were magnetically labeled. Chondrogenesis *in vitro* was assessed with histological staining using Safranin-O	Allogenic transplantation of feroxide labeled synovial MSC into osteochondral defect on articular cartilage of patellar groove of distal femur with a permanent magnet placed	Increased thickness of regenerated cartilage as compared with control, stem cells with magnets performed better than stem cells alone, increased histological scores
Mak et al. (2016)	Murine	Murine	9	43	Biopsy of synovium of knee joint	Synovial MSCs were seeded into plates and chondrogenic medium was added. qPCR was used to quantify expression of chondrogenic markers including Sox9, Col2A1, and ACAN	Allogenic intra-articular injection of MSCs into a full thickness focal cartilage defect in the femur	Increased cartilage repair in both groups, improved MRI appearance, and histological scores
Zayed et al. (2018)	Equine	Murine	Not available	Not available	Culture of sMSC derived from synovial fluid in previous experiment	sMSCs were cultured in DMEM media with TGF-β1 for 14 days. Western blot analysis was used to analyze chondrogenic differentiation	Xenogenic transplantation of fluorescently labeled MSCs encased in agarose scaffold constructs into full thickness cartilage defect in the trochlear groove of rat femur	Improved Macroscopic appearance in sMSC treated knees vs. control, sMSC treated knees demonstrated higher type II collagen expression.
Pei et al. (2010)	Porcine	Rabbit	12	12	Biopsy of intimal layer of synovium from knees joint	Synovial MSCs were culture-expanded *in vitro* and passaged once prior to seeding onto tissue engineered construct composed of polyglycolic acid (PGA). The construct was incubated for 1 month in chondrogenic growth media in a bioreactor	Xenogenic transplantation of tissue engineered construct into medial femoral condyle of surgically induced osteochondral defects	Improved defect filling in the treated group at 3 weeks by macroscopic appearance. At 6 months, worsened macroscopic appearance in the treated group as compared with the control group. Improved histological scoring in the control group compared to the treated group
Ando et al. (2007)	Porcine	Porcine	6	3	Excision of synovial membrane from knee joint	Synovial MSCs were expanded *in vitro* and cultured in chondrogenic medium containing recombinant BMP2. Immunohistology and RT-PCR were used to confirm expression of chondrogenic markers	Allogenic implantation of tissue engineered construct into chondral defects in medial femoral condyle	Improved ICRS scores in TEC implanted subjects compared to control at 6 months
Shimomura et al. (2010)	Porcine	Porcine	14	10	Biopsy of synovial membranes of knee joint	Synovial MSCs were isolated from membranes and plated in dishes with chondrogenic medium. A pellet culture system was used to assess *in vitro* chondrogenesis. RT-PCR was used to detect Collagen II expression. GAG synthesis was confirmed by Alcian blue staining	Allogenic implantation of MSCs on tissue engineered constructs into chondral defects on the medial condyle	Improved macroscopic and histological scores in treated vs. untreated groups (ICRS histological score). No difference between whether tissue was harvested from mature or immature pigs at 6 months post implantation
Chiang et al. (2014)	Porcine	Porcine	12	12	Aspiration of needle flushing fluid from synovium of knee joint	Synovial fluid was centrifuged into pellets. The pelleted cells were suspended in DMEM culture and subsequently injected into PRP composite hydrogels. Immunohistology and Real Time PCR were used to evaluate chondrogenic gene expression	Autologous implantation of MSC with platelet-rich-plasma (PRP) composite hydrogel	Improved macroscopic appearance when treated with MSC in hydrogel compared with controls (without MSC—which demonstrated degradation of the hydrogel complex) at 4 and 8 weeks follow up, greater amount of ECM deposition in treated group
Pei et al. (2013)	Porcine	Porcine	20	6	Biopsy of synovial membrane in knees of pigs	Expanded synovial MSCs were centrifuged into pellets. The pellets were cultured in chondrogenic medium and analyzed for chondrogenic differentiation at 0, 7, and 14 days following incubation. RT-PCR and immunohistology were used to examine chondrogenic differentiation	Allogenic injection of expanded cells into partial thickness cartilage defects in porcine medial femoral condyle	Improved macroscopic appearance with treated groups, greater histological scores at 3 months
Nakamura et al. (2012)	Porcine	Porcine	7	7	Biopsy of suprapatellar pouch synovium through arthrotomy of knee joint	Synovial MSCs were plated and culture expanded. The cells were transformed into pellets by centrifugation and cultured in chondrogenic medium containing DMEM, BMP7, TGF, etc. The pellets were assessed histologically with Safranin O staining	Allogenic transplantation of MSC into full thickness osteochondral defects in medial femoral condyle	Improved macroscopic appearance of defect—thicker white membrane at 2 months on arthroscopic Inspection. Significantly improved ICRS score in treated groups

Seven studies investigated sMSCs harvested from rabbit synovium, two from murine synovium, one from equine synovium, and six from porcine synovium. Three studies extracted cells from synovial fluid and 13 studies obtained cells *via* excision biopsy of the synovial membrane. All studies implanted sMSCs into femoral chondral lesions. Out of the 16 studies, 13 performed allogenic transplantation, one performed autologous and two performed xenogenic transplantation. A total of 213 subjects were treated with sMSCs. Fourteen of 16 studies showed that sMSCs were superior to control in treating full thickness chondral lesions. The method of delivering sMSCs varied among the studies.

Schmal et al. (2018) compared cultured allogenic chondrocytes with rabbit sMSCs in their ability to repair osteochondral lesions in rabbit femur. Improved macroscopic appearance was seen in the sMSC group as compared with controls up to 24 weeks post-intervention. Pei et al. (2013) employed a similar follow-up period extending to 6 months and showed that *in vitro* engineered rabbit sMSC constructs were able to regenerate rabbit chondral lesions (Pei et al., 2009). Through histological assessment, they demonstrated smooth hyaline cartilage in the treated group. Li et al. (2016) attempted to qualify the cartilage quality of repaired osteochondral lesions in rabbit knees treated with rabbit sMSC injection. Their experiment showed no significant difference in macroscopic, Modified O'Driscoll (MOD) and Magnetic resonance Observation of CArtilage Repair Tissue (MOCART) scores. However, using Deuterium Weighted Imaging (DWI) and T2 imaging settings on MRI, the group was able to reveal greater tissue quality in the treated group. Jia et al. (2018) investigated the effects of intra-articular injection of rabbit sMSCs in rabbit patellar groove osteochondral lesions by comparing predifferentiated and undifferentiated rabbit synovial fluid derived sMSCs. Predifferentiated sMSCs performed better than control groups in cartilage repair as measured by ICRS and macroscopic appearance. However, undifferentiated sMSCs were found to be superior to pre-differentiated sMSCs.

A number of studies on the rabbit model sought to investigate the effects of gels and scaffold. Lee et al. (2013) investigated platelet-rich plasma gel (PRP) for the delivery of sMSCs. Rabbit sMSCs were suspended on blood supernatant containing PRP and injected allogenically into full thickness chondral lesions. The PRP gel was observed to bind to chondral lesions and bone within 10 min following injection. At 24 weeks, the treated group demonstrated significantly improved macroscopic scores as compared with control. However, the microscopic appearances of the lesions in the treated groups were not significantly different to the untreated groups until 12 weeks post-intervention. The effects of combining different constructs were explored in a study by Shimomura et al. (2014). They merged tissue engineered construct made from sMSCs with a hydroxyapatite (HA) bone. The combined material was implanted into full thickness lesions in rabbits and was compared to a control group in which only HA was utilized. It was found that the sMSC construct integrated quickly with the HA artificial bone. The sMSC groups achieved rapid cartilage repair and demonstrated improved osteochondral appearances compared with the control group which showed changes consistent with early osteoarthritis-like features at 6 month follow-up. Lee et al. (2012) investigated the effects of intra-articular injection of rabbit sMSCs in rabbit patellar groove osteochondral lesions. The sMSCs were suspended in a collagen/hyaluronic acid/fibrinogen (COL/HA/FG) gel. This was compared to controls that received COL/HA/FG alone. The treated groups demonstrated greater Safranin-O and type II collagen staining.

Hori et al. (2011) labeled murine sMSCs with ferumoxides and treated chondral lesions by direct implantation (Hori et al., 2011). Permanent magnets were simultaneously implanted into the bottom of murine lesions. Chondral lesions treated with both labeled sMSCs and implanted magnets outperformed non-labeled sMSCs in histological scores. Cartilage thickness was also found to be greater in the labeled group. Mak et al. (2016) evaluated subsets of murine sMSCs and assessed their ability to regenerate cartilage in mice. Sca-1 positive Murphy's Roth Large mice (MRL/MpJ) derived “healer” sMSC subsets did not perform better than non-healing C57BL6 sMSC subsets. Both groups demonstrated similar cartilage repair outcomes at 4 weeks post-treatment. The MRL/MpJ group also persisted in the defect for longer than the C57BL6 subset.

Zayed et al. (2018) undertook a study to compare the ability of equine sMSCs to regenerate cartilage defects in murine models with bone marrow derived MSCs. The xenogenic transplantation study showed increased amount of type II collagen in sMSC-repaired lesions as compared with bone marrow derived MSC-treated lesions. Pei's group undertook a xenogenic transplantation study of sMSCs on a tissue engineered construct (Pei et al., 2010). sMSCs were incubated with a PGA scaffold in a bioreactor with chondrogenic growth factors for a duration of 4 weeks. Following that, the scaffolds were implanted into rabbit knee osteochondral defects and observed for 6 months. Positive results in macroscopic appearance were seen in favor of the treated group at 3 weeks. At 6 months, quantitative histological measures reveal increased tissue loss in the treated groups as compared with control groups. The control groups displayed an improved macroscopic appearance compared to the treated group.

Five separate studies examined the effects of porcine sMSCs on porcine chondral lesions. Ando et al. (2007) implanted sMSCs via a tissue engineered construct into medial condyle chondral lesion and found an improved ICRS score in implanted subjects at up to 6 months. Shimomura et al. (2010) examined the chondrogenicity of sMSCs as a function of age. They found no significant difference between mature and immature porcine sMSCs in treating osteochondral lesions in the knee. Chiang et al. (2014) explored the utility of a PRP composite gel in delivering sMSCs and similarly to Lee et al. (2012) found this to be an effective method on long term follow-up. Pei et al. (2013) allogenically transplanted *ex vivo* expanded sMSCs grown on a decolorized matrix into partial thickness lesions and observed an improvement at 3 months. Nakamura et al. (2012) looked at the effects of porcine sMSCs in treated full thickness chondral lesions when transferred allogenically via a minimally invasive approach. They found a significantly improved ICRS score and macroscopic appearance in the treated group (Nakamura et al., 2012).

## Discussion

The studies included in our review demonstrated overall positive outcomes with sMSC transplantation, and generally without complications. All but one study demonstrated significant improvement in cartilage repair as stipulated by various outcome measures when compared with controls that did not receive sMSCs. Two studies compared MSCs from synovium with MSCs from another source. Zayed's group extracted MSCs from equine joint synovium and bone marrow (Zayed et al., 2018), and performed a xenogenic transplantation study to compare the two sources in their ability to regenerate murine cartilage. Their results suggest that sMSCs have superior chondrogenic potential both *in vitro* and *in vivo*. Nakamura et al. (2012) compared sMSCs to bone marrow, muscle, periosteum and adipose derived MSCs, and found sMSCs to have greater chondrogenic potential *in vitro* (Nakamura et al., 2012).

All included studies were case-control studies on the effect of sMSC transplantation *in vivo* in humans or animal models. The studies included in this review explored a variety of different animal models. While the variation makes it difficult to draw comparisons, it does suggest sMSCs to be a robust cell source for cartilage repair. Although most studies suggest that cells isolated from different mammalian animals appear not to vary significantly in *in vitro* differentiation potential, Scuteri et al. (2014) suggest that cell harvest from murine may derive sMSCs with significantly different chondrogenicity when compared to human cell sources (Scuteri et al., 2014). It is difficult to envision future cell based therapies for human chondral lesions using xenogenic transplantation due to potential immunological implications. Pei et al.'s study of xenogenic sMSC transplantation reported poorer cartilage repair in the treated group at long term review, they suggested that this could be attributed to delayed immune rejection of the transplanted sMSCs (Pei et al., 2010). Although the studies in this review did not specifically explore these particular effects, there is some evidence to suggest that xenogenic MSC transplantation may dampen rather than trigger host T-cell responses (Ezzelarab et al., 2011).

In the studies included in our review, sMSCs were isolated either by excisional biopsy of synovial membrane or extraction of intra-articular fluid. Fülber et al. in their *in vitro* study suggested that MSCs derived from joint cavity fluid have greater *in vitro* chondrogenic potential in comparison to MSCs derived from the synovial membrane itself (Fülber et al., 2016). While there was relative consistency in method of harvest, the joint phenotypes varied significantly. This is however probably not important in humans as Koizumi et al. (2016) demonstrated that the joint phenotype from which MSCs are isolated had little effect on MSC chondrogenic potential (Koizumi et al., 2016). It is difficult to obtain significant numbers of sMSCs from direct harvest, and most studies culture-expanded sMSCs *ex-vivo* prior to transplantation. Some studies suggested that resident MSC number and chondrogenicity vary as a function of age. This may introduce difficulty in conducting reliable pooled analysis of the results. Furthermore, while the studies in this review harvested cells from joints there may still be variation in chondrogenicity.

The method of sMSC delivery varied significantly between the studies reviewed. This ranged from direct intra-articular injection, transfer on cellularized matrices, transfer on engineered acellular biomaterial scaffolds and homing by magnetism through injection of ferumoxide into MSCs (Hori et al., 2011; Jia et al., 2018; Zayed et al., 2018). Successful cartilage repair following direct injection of sMSCs may reflect the ability of synovial MSCs to home to chondral lesions. Transfer on a scaffold may control for this effect and allow more direct assessment of chondrogenic potential as the sMSCs are artificially directed to the site of the lesion. Manipulation of sMSCs by other methods, such as incorporation of ferrous metals may alter the biology of sMSCs. While this creates difficulty in direct comparison when interpreting results, it may allow for a meaningful subgroup analysis if aggregate sample sizes were adequately large. The results suggest that sMSCs are ubiquitously efficacious regardless of method of delivery and can be utilized in various forms depending on the lesion in question. These findings may also apply to MSCs from other tissue sources.

The exact mechanism for the suggested superior chondrogenicity of sMSCs compared to other cell sources have not been elucidated. Ogata suggests that it could be due to the increased prevalence of certain MSC subpopulations that carry a greater propensity to undergo chondrogenic differentiation (Ogata et al., 2015). It may be that sMSCs exhibit a greater ability to proliferate *in vitro*, and thus is less labor intensive to expand prior to transplantation. Particular studies have examined the proliferation rate of sMSCs in comparison to adipose-derived MSCs and found sMSCs to be superior (Mochizuki et al., 2006). In the same study, sMSCs were shown to have greater chondrogenic potential than adipose-derived MSCs.

As cell-based therapies for chondral defects are gaining more attention, MSCs are becoming more extensively investigated. Recently, studies have attempted to clarify the effects of MSC-derived exosomes in cartilage repair *in vitro* (Vonk et al., 2018) and *in vivo* (Tao et al., 2017; Wang et al., 2017). The authors from these studies attribute the positive effects of exosomes in cartilage repair to their modulatory effects on gene expression. *In vivo* studies exploring different delivery methods of these MSC secretomes may help identify the most effective way of translating these findings to clinical application.

One of the main limitations of this review was the heterogeneity between the studies in the methods used to assess outcomes. The objective quantitative measures of cartilage repair included macroscopic, histological, biomechanical and biochemical evaluations. The most commonly employed macroscopic scoring was the ICRS score, whereas most studies applied a unique subject score. The MOD score was the most used histological measure of cartilage repair, and was used in one-third of the studies. Macroscopic scoring systems based on defect appearance are subject to observer bias and are difficult to address. The use of a single operator could result in overestimation of therapeutic benefits, whereas multiple-operators could introduce inter-user variability (Moojen et al., 2002). In the future, the bias may be eliminated with computer algorithms delivering a reproducible assessment (Moussavi-Harami et al., 2009). Two human studies used Patient Reported Outcome Measures (PROMs) and demonstrated an improvement following sMSC treatment (Sekiya et al., 2015; Shimomura et al., 2018). While in human studies, this may be the most effective way of assessing the outcomes of MSC treatment, animals studies will need to rely on objective mechanical assessments such as gait analysis and weight-bearing distribution.

The majority of the studies in this review were at intermediate to high risk of bias as determined by the ROB-2 Cochrane risk assessment tool. For the purposes of drawing conclusions from a systematic review, an overall high risk of bias may invite caution in interpreting pooled outcomes. Whereas, an overall low risk of bias may suggest greater reliability of the conclusions drawn from a systematic review. Dissection of individual bias levels assigned to studies may help to elucidate elements lacking between studies and guide future study design. The main contributor to increased risk of bias was poor randomization processes in determining the experimental and control cohorts. Furthermore, there was significant subjectivity in certain outcome measures employed by most of the studies e.g., in histological scoring and scoring macroscopic cartilage appearance. We believe that the studies on MSCs used for cartilage repair could be improved in a number of ways including the characterization of sMSCs by identification of surface epitopes, standardization of MSC delivery methods by determining the optimal volume or cell number for a given defect size, and the use of objective quantitative measures.

## Conclusion

Regenerative approaches may be the most promising option for treating chondral lesions and preventing osteoarthritis. Cell-based therapies such as MSC transplantation are proving to be effective in *in vitro* and *in vivo* studies. The search for an optimal cell source will help guide translation to clinical application in humans. In this review we have shown that MSCs derived from synovial tissue have good chondrogenic potential. Defining the cell population being used, establishing standardized methods for MSC delivery, and the use of objective outcome measures should enable future high quality studies such as randomized controlled clinical trials to provide the evidence needed to manage chondral lesions optimally.

## Author Contributions

KT and WK were involved in the conception of the review. KT was involved in writing the manuscript and conducting data analysis in consultation with CM and WK. BZ and KR were involved in conducting a literature search and applying risk of bias analysis. All authors reviewed the final manuscript.

### Conflict of Interest

The authors declare that the research was conducted in the absence of any commercial or financial relationships that could be construed as a potential conflict of interest.
