# Supplementary material for: Synovium-Derived Mesenchymal Stem Cell Transplantation in Cartilage Regeneration: A PRISMA Review of in vivo Studies
Source: Front Bioeng Biotechnol. 2019 Nov 15;7:314. doi: 10.3389/fbioe.2019.00314 (PMC6873960; doi:10.3389/fbioe.2019.00314)
Supplement: Supplementary file 1 [file Data_Sheet_1.docx]

| Domain | Signalling question | Response |
| --- | --- | --- |
| Bias arising from the randomization process | 1.1 Was the allocation sequence random? | Yes/No/Probably/Not Applicable |
|  | 1.2 Was the allocation sequence concealed until participants were enrolled and assigned to interventions? | Yes/No/Probably/Not Applicable |
|  | 1.3 Did baseline differences between intervention groups suggest a problem with the randomization process? | Yes/No/Probably/Not Applicable |
|  | Risk of bias judgement | Low/Some Concerns/High |
| Bias due to deviations from intended interventions | 2.1.Were participants aware of their assigned intervention during the trial? | Yes/No/Probably/Not Applicable |
|  | 2.2.Were carers and people delivering the interventions aware of participants' assigned intervention during the trial? | Yes/No/Probably/Not Applicable |
|  | 2.3. If Y/PY/NI to 2.1 or 2.2: Were there deviations from the intended intervention that arose because of the experimental context? | Yes/No/Probably/Not Applicable |
|  | 2.4 If Y/PY to 2.3: Were these deviations likely to have affected the outcome? | Yes/No/Probably/Not Applicable |
|  | 2.5. If Y/PY/NI to 2.4: Were these deviations from intended intervention balanced between groups? | Yes/No/Probably/Not Applicable |
|  | 2.6 Was an appropriate analysis used to estimate the effect of assignment to intervention? | Yes/No/Probably/Not Applicable |
|  | 2.7 If N/PN/NI to 2.6: Was there potential for a substantial impact (on the result) of the failure to analyse participants in the group to which they were randomized? | Yes/No/Probably/Not Applicable |
|  | Risk of bias judgement | Low/Some Concerns/High |
| Bias due to missing outcome data | 3.1 Were data for this outcome available for all, or nearly all, participants randomized? | Yes/No/Probably/Not Applicable |
|  | 3.2 If N/PN/NI to 3.1: Is there evidence that result was not biased by missing outcome data? | Yes/No/Probably/Not Applicable |
|  | 3.3 If N/PN to 3.2: Could missingness in the outcome depend on its true value? | Yes/No/Probably/Not Applicable |
|  | 3.4 If Y/PY/NI to 3.3: Is it likely that missingness in the outcome depended on its true value? | Yes/No/Probably/Not Applicable |
|  | Risk of bias judgement | Low/Some Concerns/High |
| Bias in measurement of the outcome | 4.1 Was the method of measuring the outcome inappropriate? | Yes/No/Probably/Not Applicable |
|  | 4.2 Could measurement or ascertainment of the outcome have differed between intervention groups? | Yes/No/Probably/Not Applicable |
|  | 4.3 Were outcome assessors aware of the intervention received by study participants? | Yes/No/Probably/Not Applicable |
|  | 4.4 If Y/PY/NI to 4.3: Could assessment of the outcome have been influenced by knowledge of intervention received? | Yes/No/Probably/Not Applicable |
|  | 4.5 If Y/PY/NI to 4.4: Is it likely that assessment of the outcome was influenced by knowledge of intervention received? | Yes/No/Probably/Not Applicable |
|  | Risk of bias judgement | Low/Some Concerns/High |
| Bias in selection of the reported result | 5.1 Were the data that produced this result analysed in accordance with a pre-specified analysis plan that was finalized before unblinded outcome data were available for analysis? | Yes/No/Probably/Not Applicable |
|  | 5.2 ... multiple eligible outcome measurements (e.g. scales, definitions, time points) within the outcome domain? | Yes/No/Probably/Not Applicable |
|  | 5.3 ... multiple eligible analyses of the data? | Yes/No/Probably/Not Applicable |
|  | Risk of bias judgement | Low/Some Concerns/High |
| Overall bias | Risk of bias judgement | Low/Some Concerns/High |

Supplementary Table 1. RoB 2.0 signalling questions
